# First Report on Evaluation of Basic Nutritional and Antioxidant Properties of *Moringa Oleifera* Lam. from Caribbean Island of Saint Lucia

**DOI:** 10.3390/plants8120537

**Published:** 2019-11-23

**Authors:** Jozef Fejér, Ivan Kron, Vito Pellizzeri, Mária Pľuchtová, Adriana Eliašová, Luca Campone, Teresa Gervasi, Giovanni Bartolomeo, Nicola Cicero, Andrea Babejová, Mária Konečná, Vincent Sedlák, Janka Poráčová, Daniela Gruľová

**Affiliations:** 1 Department of Ecology, Faculty of Humanities and Natural Sciences, University of Prešov, 17 Novembra 1, 08116 Prešov, Slovakia; jozef.fejer@unipo.sk (J.F.); maria.pluchtova@gmail.com (M.P.); adriana.eliasova@unipo.sk (A.E.); 2 JNT Ltd., 04001 Košice, Slovakia; kron.ivan@gmail.com; 3 Department of Biomedical and Dental Sciences and Morphofunctional Imaging, University of Messina, 98122 Messina, Italy; vpellizzeri@unime.it (V.P.); tgervasi@unime.it (T.G.); gbartolomeo@unime.it (G.B.); nicola.cicero@unime.it (N.C.); 4 Laboratory of Food Chemistry, Dipartimento di Agraria (QuaSic. A. Tec.), University of Mediterranea di Reggio Calabria, 89124 Reggio Calabria, Italy; lcampone@unisa.it; 5 Department of Biology, Faculty of Humanities and Natural Sciences, University of Prešov, 17 Novembra 1, 08116 Prešov, Slovakia; maria.konecna@unipo.sk (M.K.); janka.poracova@unipo.sk (J.P.)

**Keywords:** Caribbean, hydroxyl radical, lipids, moringa, neohesperidin, proteins, phenols, quercetin, superoxide radical, vitamin E

## Abstract

*Moringa oleifera* Lam. has been considered as a multipurpose tree. The studies on it focus on its variable nutritional benefits. It is growing in many regions, but information about nutritional properties of those growing in the Caribbean is missing. The present study focused on biochemical analysis of main nutritional and antioxidant properties in plant material—dried leaves and seeds—of *Moringa oleifera*. The composition of lipids, proteins, and vitamin E was evaluated in powdered dried leaves and seeds. Fatty acids were evaluated in oil extracted from the moringa seeds. Potential antioxidant properties of the moringa were evaluated in extract from crushed and powdered leaves, as well as from the powdered seeds. The total amounts of lipids, proteins, and vitamin E were higher in powdered seeds (31.85%, 35.13%, and 220.61 mg/kg) than in powdered leaves (12.48%, 20.54%, and 178.10 mg/kg). The main compound of fatty acids presented oleic acid (76.78%) in seeds’ oil and oleic (25.01%), palmitic (24.84%), and linolenic (24.71%) acids in leaves. Neohesperidin (126.8 mg/kg), followed by chlorogenic acid (99.96 mg/kg) and quercetin (43.44 and 21.44 mg/kg) were main phenolic compounds identified. Total phenols in powdered leaves’ extract (635.6 mg GAE/L) was higher than in powdered seeds’ extract (229.5 mg GAE/L). The activity against superoxide radical and hydroxyl radical was 92.4% and 73.1% by leaves’ powder extract and 83.6% and 60.7% by crushed-leaf extract; seed-powder extract exhibited a pro-oxidation activity (−68.4%) against superoxide radical and the lowest antioxidant effect against the hydroxyl radical (55.0%).

## 1. Introduction

*Moringa oleifera* Lam. (moringa tree) belongs to the *Moringaceae* family [1]. It originates from the Himalayan Mountains in North India and Pakistan and is spread widely in tropical and subtropical areas of Africa, the southeastern part of Asia, and South America [2,3,4]. It is commonly cultivated as a vegetable in different countries, including Pakistan, Thailand, and the Philippines [5]. *M. oleifera* has been commercially expanded to Mexico, Hawaii, Cambodia, and the Caribbean Islands [6,7]. *M. oleifera* is considered to be a multipurpose tree [5]. All parts of the plant can be utilized by humans [8]. Various reviews noted antioxidant, antimicrobial, anti-inflammatory, anticancer, analgesic, cardiovascular, antiulcer, immunomodulation, antihypertensive, hepatoprotective, diuretic, antiurolithiatic, anthelmintic, hypoglycemic, antidiabetic, anti-asthmatic, and antiaging potentials of the plant [1,9,10,11,12,13,14,15,16,17]. Its benefits in nutritional value present higher standard in dietary intake than is recommended by the WHO [5]. *M. oleifera* was also used in ancient times in India. One record revealed that emperors ate its leaves and fruits to keep their skin healthy and mind sharp, and the warriors ate the extract of leaves during the war to get more energy and relieve tension and pain [18]. Based on its specific chemical composition, this plant is considered as nutraceutical food [19]. In spite of its enormous properties and broad range of uses, *M. oleifera* is still unexploited, especially in countries outside the origin areas [20]. There have been published articles about the nutritional and health benefits of the *Moringa* species. All of these studies focused on plant material grown and collected mostly in the countries of Asia, particularly India, or Africa. No publications have evaluated the nutritional values of *M. oleifera* in Caribbean.

Our interest focused on specific products, which include “exotic” moringa powder. These products have flooded the European market during the last two years. There are a lot of food supplements that have demonstrated the nutritional and antioxidant value of moringa powder, as well as a lot of cosmetics products with antiaging properties. Moringa leaves have been reported to be a rich source of β-carotene, proteins, vitamin C, calcium, and potassium, and they act as a good source of natural antioxidants [21]. A variety of nutritional values, as well as various methods like FRAP and DPPH, which are used to study antioxidant activity of moringa, were used in present study. The antioxidant capacity is a property of several classes of organic compounds present in various type of natural products, such as polyphenols in wine [22], carotenoids produced by microbial activity [23], or squalene present in olive oils and pistachio [24,25]. Meat, beans, and single-cell protein presence various sources of proteins are not all good for human supply [26]. New alternative sources of proteins are the point of interest.

To the best of our knowledge, this is the first study which investigated the various chemical compositions with nutritional potential and antioxidant activity of *M. oleifera* from the Caribbean region. There have been evaluated phenolic compounds and total phenols, lipids, proteins, vitamin E, and antioxidant activity from leaves and seeds, compared with the previous studies on the same species from different world regions.

## 2. Results

### 2.1. Phenols Components

Based on the described method, nine phenolic compounds in moringa extract from leaves and seven phenolic compounds in seeds’ extract were identified. The dominant phenolic compound identified was neohesperidin (126.8 mg/kg), followed by chlorogenic acid (99.96 mg/kg) and quercetin (43.44 mg/kg) in leaves extract, and quercetin (21.44 mg/kg) and p-hydroxybenzoic acid (2.66 mg/kg) in extract from seeds (Table 1).

### 2.2. Vitamin E, Lipids, and Proteins

The amounts of vitamin E, lipids, and protein were determined in moringa leaves and seeds. (Table 2). In all three assays, the higher amount was noted in extract from seeds compared to the extract from leaves. The quantity of vitamin E in leaf extract was 178.10 mg/kg, and, in seed extract, it was 220.61 mg/kg. The amount of lipids was more than doubled in seed extract (31.85%) in comparison with leaf extract (12.48%). Some differences were observed in quantifying the amount of proteins in both extracts, where 20.54% was calculated in leaf extract and 35.13% in seed extract.

### 2.3. Fatty Acids

Thirteen fatty acids were identified in the extracts from leaves and seeds. The highest content in seed extract presented oleic acid as 76.78%. Very low quantities of essential fatty acids were found in moringa extract from seeds, where linoleic acid presented 0.62% and linolenic acid only 0.13%. Dominant fatty acids were identified as oleic (25.01%), palmitic (24.84%), and linolenic (24.71%) acids in leaf extract. The ratio between saturated (38.9%), monounsaturated (27.52%), and polyunsaturated (33.51%) fats in leaf extract was almost balanced. The comparison of our findings with other results [27] is shown in Table 3. While saturated fatty acids were similar in leaves, monounsaturated fatty acids from seeds were more than six times higher in our sample, and polyunsaturated fatty acids in our sample were almost half of the compared source.

### 2.4. Antioxidant Activity

The results of the dry-matter extracts, total phenol content, and antioxidant activity against hydroxyl and superoxide radicals are shown in the Table 4. In the cases of phenol content and antioxidant activities, we included the conversion to dry-mass amounts for the extracts. Such values much better express the relative antioxidant activity related to the content of phenols, which are different in the plant extracts. The amount of dry matter in the extracts depended on the plant material used. The highest amount of the dry matter was found in the crushed-leaf extracts, and the lowest content was found in the seed-powder extract. A statistically highly significant difference was found between the dry-matter content of leaf extract and seed extract (*p* < 0.001). The yield obtained from three different samples of moringa plant material was in the range 2.1–2.8%. The content of total phenolic substances depended on the moringa specimen extract. The highest content was found in leaf extract 635.6 mg GAE/L, or 23.7 mg GAE/g DM respectively. The lowest amount was found in the seed powder extract, which was 229.5 mg GAE/L, or 11.1 mg GAE/g DM, respectively. These differences in total phenols were statistically highly different among the evaluated extracts (*p* < 0.001). The amount of phenols in moringa (seeds and leaves) extracts varies from approximately 1% to 3% of DM.

A difference in phenolic content between leaf powder and crushed leaves prepared by different methods (23.7 vs. 15.6 mg GAE/g DM, respectively) was observed. The highest activity against superoxide radicals was found in leaf extract: 92.4%, or 3.4%/g DM, respectively. Slightly lower activity was observed in the crushed-leaf extract: 83.6% or 3.0%/g DM, respectively. In contrast, seed-powder extract exhibited a relatively high prooxidative activity of −68.4% or −3.3%/g DM, respectively. The sodium salicylate standard, which was included as a control, also had a prooxidative activity of −14.1%. The variance analysis showed a statistically significant difference between extracts obtained from leaf powder and crushed leaves compared to both standard and seed-powder extract (*p* < 0.001). There was no statistically significant difference in antioxidant activity between leaf extracts and crushed leaves. Antioxidant activity against superoxide radicals is based on the transfer of electron/s to the antioxidant molecule (usually phenols). The powdered-leaf extract also had the highest activity against the hydroxyl radicals of 73.1%, or 2.7%/g DM, respectively. The crushed-leaf extract showed 60.7% activity, or 2.2%/g DM, respectively. For seed-powder extract, activity was the lowest, at 55.0%, or 2.7%/g DM, respectively. Differences in antioxidant activity were statistically significantly higher for all types of extracts in comparison to the gallic acid standard for which the anti-hydroxyl radical activity of 44.96% was recorded. Also, a significantly higher activity of leaf-powder extract was calculated in comparison to other extracts. Corrected values by DM content showed no difference between powdered-leaf extract and seed-powder extract antioxidant radicals. Both had higher activity than the crushed-leaf extract.

**Figure 1:**
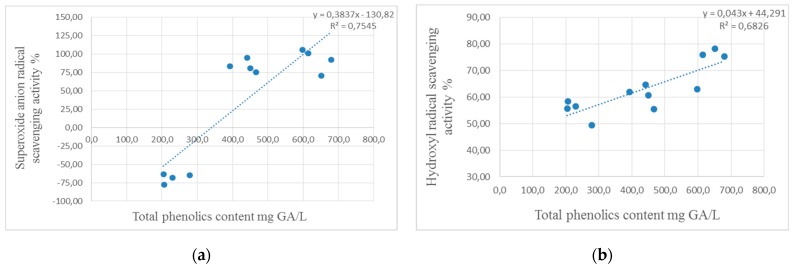
The correlation analysis demonstrated the effect of (**a**) total phenolic compounds on superoxide anion radical scavenging activity and (**b**) hydroxyl radical scavenging activity.

High correlation coefficients reveal strong antioxidant activities of phenolic compounds in ethanol extracts of moringa specimens against both tested radicals—superoxide anion and hydroxyl (Figure 1).

### 2.5. Content of Minerals, Vitamins, and Amino Acids

Dried plant material of *Moringa oleifera* was subjected for analysis of minerals, vitamins, and amino acids, in an accredited laboratory. The content of minerals are as follows: magnesium (Mg) 1618.7 mg/kg; copper (Cu) 5.9 mg/kg; phosphorus (P) 2067.0 mg/kg; zinc (Zn) 30.6 mg/kg; iron (Fe) 57.7 mg/kg; and calcium (Ca) 1,2567.0 mg/kg (Table 5).

Based on the analysis of some vitamins, there was evaluated content of vitamin C (ascorbic acid) in the amount of 3210.0 mg/kg; content of B2 (riboflavin) was 4.0 mg/kg; content of vitamin A (retinol acetate) was identified in amount of 10.2 mg/kg; and content of vitamin PP (nicotinamide) in amount of 34.0 mg/kg (Table 6).

We also analyzed individual amino acids in the dried plant material of *Moringa oleifera*. The 18 identified amino acids were aspartic acid in amount of 23.2 g/kg; threonine 10.0 g/kg; serine 6.9 g/kg; glutamic acid 33.8 g/kg; proline 11.9 g/kg; glycine 14.0 g/kg; alanine 16.1 g/kg; valine 15.4 g/kg; isoleucine 12.5 g/kg; leucine 22.9 g/kg; lysine 15.8 g/kg; arginine 21.4 g/kg; tyrosine 7.8 g/kg; phenylalanine 14.4 g/kg; histidine 7.1 g/kg; cysteine 3.5 g/kg; methionine 4.2 g/kg; and tryptophan 5.4 g/kg (Table 7).

## 3. Discussion

Flavonoids as secondary metabolites with several metabolic functions could be considered as the main phenolic compounds in moringa plants [28]. Another study [29] reported that twelve flavonoids, including quercetin, kaempferol, and apigenin, presented dominant phenolic compounds in seven cultivars of *M. oleifera* grown in Pakistan. Furthermore, a predominant group of phenolic compounds in *M. oleifera* leaves from Madagascar were identified as the flavonoid group with kaempferol and quercetin [30]. However, it is important to consider that phenolic compound yields are strongly dependent not only on the season, the weather conditions, and the application of fertilizers, but also on the cultivar and genetic variability, which could be the most relevant factors for the phytochemical composition of moringa leaves [29]. These reports differ from our findings, where the dominant compounds were neohesperidin and chlorogenic acid, and after them followed quercetin in leaves, while, in seeds, quercetin was evaluated as a major compound.

Three derivatives of tocopherols (α, γ, and δ) were determined at 15.38, 4.47, and 15.51 mg/kg, respectively in *M. oleifera* seeds oil (variety Periyakulam 1) from India [31]. The higher amounts of 98.82, 27.90, and 71.16 mg/kg of α, γ, and δ-tocopherols, respectively, have been found in the different variety Mbololo from Kenya [32]. In the sample of *M. oleifera* from Pakistan were identified tocopherols (α, γ, and δ) in contents of 140.5, 63.18, and 61.70 mg/kg [33]. The determined quantity of vitamin E in our samples were 178.1 mg/kg in leaves and 220.6 mg/kg in seeds.

Lipid content in the range from 12.5% to 21.6% had been found in moringa dried leaves from three localities in Burkina Faso [34]. Lower content was determined in the sample from Ethiopia—about 10% [35]. Low content of lipids in dry leaves and leaf powder (5.2% and 2.3%), compared to *M. oleifera* seeds (38.7%), were previously described [36]. The amount of lipids in the samples from Caribbean was 12.45% in leaves and more than doubled (31.85%) in seeds.

It has been reported that the protein content in moringa leaves and seeds ranges from 22 to 36.7 g/100 g of DW [29,36,37,38]. The quantity of determined crude proteins in dry leaves of *M. oleifera* from South Africa was 30.3% [27]. However, maximum protein content (37.5 g/100 g of DW) was recently reported for *M. oleifera* seeds cultivated in Mexico [39]. It has been suggested that the protein quantity depends not only on the cultivars and species but also on environmental factors. Very little differences in the amount of the protein in leaf extracts were determined in two regions in Ghana: reached 26.54% and 25.65% [40]. Other studies have reported variable protein contents ranging between 16% and 40% [4,19,34,41,42,43,44]. The mean protein content found in the Ethiopia samples from a market of Mekelle in ranged from 10.74% to 11.48% [35]. The content of protein in Caribbean samples was identified within the range in mentioned previous studies.

The content of principle fatty acid, i.e., oleic acid, was well in line with that reported for *M. oleifera* oil from Kenya [32] and India [31] (Lalas and John Tsaknis, 2002). High content of oleic acid (65.00%) was determined in moringa seed extract from Saudi Arabia [45]. The dried moringa leaves from South Africa contained 17 fatty acids, and α-linolenic acid (44.57%) had the highest value, followed by heneicosanoic (14.41%) [27]. Fatty acids, namely, oleic, palmitic, heptadecanoic, stearic, arachidic, linoleic, linolenic, eicosenoic, and behenic acids, among others, were identified as the main components of moringa oil, according the previous publications [19,38,46,47]. Monounsaturated fatty acids, such as palmitoleic (C16:1), oleic (C18:1), and eicosenoic (20:1) acids, are present in the largest amounts [46,48]. The composition of M. oleifera seed oil fatty acid reveals that it falls in the category of high-oleic oils (C18:1, 67.90–76.00%) [21]. Conjugated linoleic acid has attracted increased research interest because of its health-promoting benefits and biological functions [49]. Oleic acid is the most prevalent unsaturated acid in *M. oleifera* seed oils, which can be viewed as a healthy alternative to partially hydrogenated vegetable oils [38,47]. Therefore, due to their high unsaturated fatty acid content, moringa seed oils are ideal substitutes for olive oils [36].

Extract from moringa flowers obtained by a similar procedure gave 8.7% yield [50]. Much higher yield (18.8%) was obtained by [8], who extracted crushed leaves with a methanol–water mixture (80:20) for three days. The effect of various methods of moringa leaves’ drying followed by extraction with water (the ratio 1 g of leaves to 100 ml distilled water) for 24 h, yielded approximately 44% for each sample [51]. Another study reported the amount of phenols in methanol extracts of moringa leaves from India as 118 mg/g, which is almost 12% [52]. They used a different solvent and different ratio between plant material and solvent. The strong effect of moringa leaves’ drying methods on total phenolics (and other compounds, as well) was shown by [51]. They found from 47 to 69 mg GAE/g DM. Compounds exposed to the superoxide radicals had a longer time change from antioxidant to prooxidant activity [53,54], so antioxidants protect the tissues/cells from damage by radicals, on other side, when they turn to prooxidants that can dispose of cancer cells [1]. Antioxidants (phenols and other molecules) can act against hydroxyl radicals in different ways in comparison to superoxide radicals. They incorporate hydroxyl radicals faster and more easily into their structure and prevent the damage to more vulnerable molecules. This ability depends indirectly on the number of substituents in phenolic molecules.

Knowledge of human micronutrient requirements is a crucial step in understanding the public health significance of micronutrient malnutrition and identifying the most appropriate measures to prevent them. Essential vitamins and minerals include vitamins A, C, D, E, and K; the B vitamins; calcium; iron; magnesium; zinc; selenium; and iodine [55]. Vitamins are organic compounds that are needed in small quantities to sustain life. Most vitamins need to come from food. Different vitamins have different roles, and they are needed in different quantities [56]. According to WHO and FDA, the Daily Values are the amounts of nutrients recommended per day for humans who are four years of age or older. The following are requirements for selected vitamins and minerals: vitamin A 270–300 μg RE*/D (RE = retinol equivalent per day); riboflavin (B_2_) 1.7 mg; vitamin C 60 mg; vitamin E 30 IU, vitamin K 80 μg; Ca 1000 mg; Cu 2 mg; Fe18 mg; Mg 400 mg; P 1000 mg; and Zn 15 mg [55].

Dietary requirement is the amount of protein or its constituent amino acids, or both, that must be supplied in the diet, in order to satisfy the metabolic demand and achieve nitrogen equilibrium [57]. Proteins are long chains of amino acids that form the basis of all life. The structure and function of human bodies depend on proteins. The regulation of the body’s cells, tissues, and organs cannot happen without them. Twenty amino acids can be arranged in millions of different ways, to create millions of different proteins, each with a specific function in the body. The structures differ according to the sequence in which the amino acids combine [58]. The FDA recommends that adults consume 50 grams of protein a day, as part of a 2000-calorie diet. Total indispensable amino acid recommended by FAO/WHO/UNU is 93.5 mg/kg per day [59]. The individual amino acid daily intake, as recommended, is lysine in amount 30 mg/kg; leucine 39 mg/kg; isoleucine 20 mg/kg; valine 26 mg/kg; threonine 15 mg/kg; tryptophan 4 mg/kg aromatic amino acid (phenylalanine and tyrosine) in amount of 25 mg/kg; and sulfur amino acids 15 mg/kg [57].

## 4. Materials and Methods

### 4.1. Chemicals

All standards of polyphenols, methanol (MeOH), and water HPLC grade were purchased from Sigma-Aldrich (Bellefonte, USA) and Extrasynthese (Lyon FRANCE). Syringe filters (0.2 µm PTFE) were obtained from Supelco (Milan, Italy). The formic acid and ammonium formate were purchased from Fluka (Steinheim, Germany). The individual polyphenols standard solution (1mg/mL) in pure methanol was stored at −20 °C. The mix of polyphenols standard solutions ranged from 1.0 to 50.0 mg/L and was made by appropriate dilutions of the stock solution (200 mg/L) with methanol/water (60:40) and stored at −20 °C.

### 4.2. Plant Material and Extract Preparation

Dried plant material (leaves and seeds) were received directly from a grower of *Moringa oleifera* Lam., from the Caribbean island Saint Lucia (from company Moringa Caribbean s.r.o.), and stored as plant voucher under the number M.C.321, at the Department of Ecology, University of Prešov. Ten grams of seed powder, 10 g of leaf powder, and 10 g of crushed leaves, each separately, were dissolved in 100 mL of 70% ethanol. The extraction was carried out for 72 hours at room temperature with occasional shaking. The obtained extracts were filtered over filter KA 1-M (very fast) (Papírna Perštejn s.r.o., Perštejn, Czech Rep.). The dry matter (DM) content was determined in the filtrates.

### 4.3. Phenolic Compounds Extraction

One gram of dried-leaf powder and dried-seed powder was added to 10 mL of mixture MeOH/H_2_O (80:20), and then it was stored in ultrasonic bath for 30 min. After this procedure, the samples were centrifuged at 5000 rpm for 5 min. The supernatant was collected into the flask. The extraction procedure was repeated for three times. The liquid solution of this procedure was evaporated by a vacuum system, under 35 °C, in a rotate evaporator (Büchi, Donau Lab., Flawil, Switzerland). Obtained raw extract was dissolved in 2 mL of mixture MeOH/H_2_O (60:40). In order to perform the method optimization, three mixtures of solvent were used, MeOH/H_2_O (80:20), EtOH/H_2_O (80:20), and Acetone/H_2_O (80:20), with two different times of extraction (three times for 1 h and three times for 30 min). All tests were conducted at room temperature (25 °C), in order to prevent the degradation of phenolic compounds. Validation of extraction procedure was carried out by measuring the single polyphenols amount in two samples of moringa leaf powder spiked with a mix of polyphenols, at two different concentration levels (10 and 20 mg/L).

### 4.4. UFLC-MS Determinations

The content of phenols in moringa extracts were carried out on ultra-fast liquid chromatograph, (UFLCUXR) combined with an LCMS-8040 (Shimadzu, Kyoto, Japan). The UFLC was equipped with an auto sampler (SIL-20A XR), a Kinetex C18 column (100 × 2.1mm, 1.7μm) supplied by Phenomenex (Torrance, CA, USA). The LCMS-8040 triple-quadrupole mass analyzer was equipped with electrospray ion source (ESI). The MS system was operated in electrospray negative mode at the conditions described in [60]. The analysis was conducted in MRM acquisitions modes. The mobile phase composition consisted of water with formic acid 0.1% (eluent A) and MeOH with formic acid 0.1% (eluent B), at a flow rate of 0.3 mL/min. The gradient was start 0–7.00 min 2% B, 7.00–10.50 min 17% B, 10.50–16.00 min 17% B (isocratic), 16.00–23.00 min 100% B, 23.00–26 min 100% B (isocratic), 26.00–26.20 min 2% B, and 26.20–30.00 min 2% B (isocratic).

Data were analyzed with Lab solution LCMS software (version 5.53 SP2, Shimadzu, Japan). Identification was based on MS/MS transitions and retention time of target phenolic compounds. Prior to injection, samples of moringa extracts (aliquot 500 µL) were added with 100 µL of internal standard at concentration of 102.7 mg/L and 400 µL of mixture MeOH/H2O (60:40); each sample’s analysis was performed in triplicate. Calibration curves of the phenolic compounds determined in tested extracts were built-up on five different concentration levels, using Benzoic Acid as internal standards, in the linear range 1.0–50.0 mg/L. Each point of the curve was derived from triplicate injections. Coefficients of linear regression (R2) were on average 0.9997. Validation of method was carried out in accordance with Eurachem Guidelines [61]. Methanol extract of moringa leaf powder (0.9 mL), which was obtained after polyphenolic extraction, was spiked with 0.1 mL of standard mix at 10 mg/L, to have a final concentration level of 1 mg/L. This solution was injected 10 times consecutively; LOD values were obtained by multiplication of SD (standard deviations measured during the 10 injections) for 3.3 instead, and the LOQ values were determined by multiplication the LOD values for 3.

### 4.5. Preparation of Extracts for Vitamin E Determination

The extracts of moringa for vitamin E determination were prepared by using powder from leaves and seeds. Ten grams of each sample was put into falcon tubes, and 10 mL of n-hexane was added. The mixture was shaking for one minute and then was placed into ultrasonic bath for 30 minutes. Thereafter, the extracts were centrifuged at 5000 rpm for 5 minutes, and supernatant was collected into a flask. The extract obtained with this mode was ready for vitamin E analysis.

### 4.6. Determination of Vitamin E (α-tocopherol)

Chromatographic analysis of α-tocopherol was carried out by UFLC-FLR (Shimadzu) equipped with DGU20A degasser system, LC-20AD quaternary pump, CTO-20A column oven, and manual injection valve with 20 µL loop, RF-20A fluorometric detector set at 290 nm of excitation wavelength and 330 nm emission wavelength. The column used for analysis was LiChroCART 250-4 LiChrosorb Si 60 (5µm) 250 mm × 4.6 mm (Merk Millipore, Darmstadt, Germany). The analysis was performed in isocratic mode by using mobile phase n-hexane/ethyl acetate (9:1) at 40 °C, in 10 minutes. Identification of vitamin E was based on retention time of α-tocopherol. Calibration curve for quantification of α-tocopherol was obtained by injection of four standard solutions at different concentrations in the range from 1.66 to 83.33 mg/kg with R2 = 0.9992. The analysis of sample was performed in triplicate.

### 4.7. Lipids Extraction and Determination

The lipids extraction was carried out according to the ISO 659 official method. The 10 g powdered samples were extracted with 150 mL of n-hexane (Sigma-Aldrich, Milan, Italy) in a Soxhlet apparatus, for 8 h. The solvent was partly removed in a rotary vacuum evaporator (Büchi, Donau Lab., Switzerland), the residue was transferred in a pre-weight glass vessel, and the rest of the solvent was removed under nitrogen stream (99.9990% purity), to a constant weight, in order to determine the lipids content (ISO 659 2009).

### 4.8. Fatty Acids Profile Determination

The profile of fatty acid was determined by GC-FID, according to the method described in [62].

### 4.9. Content of Proteins

Nitrogen content was determined by the Kjeldahl method [63], and the total nitrogen content was multiplied by 5.75, to determine total protein. The test was performed in triplicate.

### 4.10. Superoxide Anion Radical Scavenging Activity

The assay was performed according to standard method [64] with slight modifications. Phosphate buffer (PB, sodium dihydrogenphosphate dihydrate and disodiumhydrogen phosphate dodecahydrate) 0.05 mol/L pH 7.4 was used with 0.1 mmol/L Na_2_EDTA. Hypoxanthine (HX, Alfa Aesar a Johnson Matthey Company) 0.4 mmol/L was dissolved in the phosphate buffer. Then, 0.01 g of xanthine oxidase (XO, Sigma Aldrich, activity 0.11 units/mg solid or 0.713 units/mg proteins) was dissolved in 20 mL of phosphate buffer. Nitro blue tetrazolium chloride (NBT, Sigma Aldrich) 5 mmol/L in phosphate buffer was used as an indicator of superoxide radicals. Antioxidant activity of tested samples against superoxide radicals was compared with antioxidant activity of salicylic acid (SA) 10 mmol/L in the phosphate buffer. The solutions were pipetted (volumes are in µL) into test tubes (in duplicates), according to the Table 8.

Solutions were mixed well and incubated in a water bath at 38 °C for 40 minutes. After incubation and cooling, the absorbance of the solutions was determined in 1 cm cuvette at 560 nm, using the spectrophotometer Shimadzu UV-1800. The antioxidant activity of each sample, expressed as percentage of inhibition (POI), was calculated by the following formula:

(1) $$POI\;=\;\frac{\left\{\left[A\left(X0\right)\;-\;A\left(0\right)]\;-\;[A\left(Sx\right)\;-\;A\left(0-Sx\right)\right]\right\}\times 100}{\left[A\left(X0\right)\;-\;A\left(0\right)\right]}$$

All determinations of antioxidant activity against superoxide radical in the samples were performed at least four times.

### 4.11. Hydroxyl Radical Scavenging Activity

The assay was performed according to standard method [65]. Phosphate buffer (PBS, NaH_2_PO_4_/Na_2_HPO_4_) 0.05 mol/L pH 7.4 was used with 0.1 mol/L of NaCl and 9 mmol/L 2-deoxyribose. Then, 3 mmol/L ferrous sulfate heptahydrate was dissolved in 100 mL DDW (double-distilled water) with addition of 0.1 mL concentrated sulfuric acid, to prevent oxidation of Fe(II) and hydrolysis of Fe(III). Hydrogen peroxide 10 mmol/L was dissolved in 100 mL of DDW with addition of 0.1 mL concentrated sulfuric acid, to prevent disproportionation of hydrogen peroxide. Thiobarbituric acid (TBA) 1 g was dissolved in 100 mL of 50 mmol/L sodium hydroxide solution. Trichloroacetic acid (TCA) 5.6 g was dissolved in 100 mL of DDW. Antioxidant activity of tested samples against hydroxyl radicals generated by the Fenton reaction was compared with antioxidant activity of gallic acid (GA) 10 mmol/L in the PBS. The solutions were pipetted (volumes are in µL) into test tubes (in duplicates), as shown in Table 9.

Solutions were mixed well and incubated in a water bath at 38 °C for 40 minutes. Then, 500 µL of TBA solution was added into each test tube, stoppered, mixed well, and incubated in a boiling water bath for 10 min. After boiling and uncorking, 500 µL of TCA solution was added into each test tube, mixed well, and cooled down in a beaker, with tap water. The absorbance of the solutions was determined in 1 cm cuvette at 532 nm against DDW using the spectrophotometer Shimadzu UV-1800. The antioxidant activity of each sample, expressed as POI, was calculated by the formula:

(2) $$POI=\frac{\left\{A\left(100\right)\;-\;\left[A\left(Sx\right)\;-\;A\left(0-Sx\right)\right]\right\}\times 100}{A\left(100\right)}$$

All determinations of antioxidant activity against hydroxyl radical in the samples were performed at least four times.

### 4.12. Total Phenols

The total phenolic content of the ethanol extracts of leaves and seeds was determined with the Folin-Ciocalteu reagent (FCR, Merck), according to the described procedure [66], with slight modifications. The working solution was prepared by mixing 1 volume of FCR with 9 volumes of 5% sodium carbonate solution. The solutions were pipetted (volumes are in µL) into test tubes (in duplicates), as shown in the Table 10.

Solutions were mixed well and incubated in a water bath at 40 °C for 20 minutes. Then, the absorbance of solutions was determined at 765 nm with the spectrophotometer Shimadzu UV-1800. The amounts of polyphenols in the samples were calculated as Gallic acid equivalents (GAE). All determinations of total polyphenols in the samples were performed at least four times. All solutions were used on the day of preparation.

### 4.13. Minerals, Vitamins, and Aminoacids

Six minerals (Mg, Cu, P, Zn, Fo, and Ca), four vitamins (vitamin C—ascorbic acid, vitamin B2—riboflavin, vitamin A—retinol acetate, and vitamin PP—nicotinamide), and 18 amino acids were additionally analyzed by AES-ICP (atomic emission spectrometry with inductively coupled plasma), HPLC-DAD (high-performance liquid chromatography system with diode array detector), and LC-ionex (liquid chromatography type ionex), in an accredited laboratory El spol. s r.o., Spišská Nová Ves (Slovakia), accredited testing laboratories according ISO/IEC 17025, SNAS.

### 4.14. Statistical Analysis

The statistical software Statgraphics 5.0 and multifactorial analysis of variance (ANOVA), method LSD (Least significant differences) 95% were used for statistical analysis. 

## 5. Conclusions

*Moringa oleifera* Lam. is a fast-growing tree with interesting benefits for human health. It is traditionally cultivating in its origin region, India, as well as in near Asian countries. *Moringa* was also introduced to other tropical regions as an interesting agricultural crop. Our investigation in evaluation basic nutritional and antioxidant activity of *M. oleifera* cultivated in the Caribbean island of St. Lucia showed little differences in comparison with the same species cultivated in different regions. Its various biological activities need to be studied in addition to its benefits, which are already known. Another investigation is required to confirm possible chemical biodiversity within the mentioned species based on regional deployment.

## Figures and Tables

**Table 1:** Comparison of the number of phenolic components in moringa leaves and seeds extract.

Compound	Amount ± SD (mg/kg)	
	Leaves	Seeds
p-hydroxybenzoic	5.16 ± 0.21	2.66 ± 0.12
protocatechuic acid	9.92 ± 0.32	tr
caffeic acid	1.52 ± 0.07	tr
p-cumaric acid	tr	tr
quercetin	43.44 ± 1.23	21.48 ± 0.92
gallic acid	tr	tr
hydroxytyrosol	4.22 ± 0.22	
neohesperidin	126.80 ± 2.45	tr
chlorogenic acid	99.96 ± 1.98	

Data represent the mean ± SD (standard deviation); tr = traces.

**Table 2:** Determined quantity of vitamin E, lipids, and proteins in moringa extracts from seeds and leaves.

Amount ± SD			
	Vitamin E (mg/kg)	Lipids (%)	Proteins (%)
leaves	178.10 ± 1.55	12.48 ± 0.62	20.54 ± 0.85
seeds	220.61 ± 1.80	31.85 ± 1.54	35.13 ± 1.25

Data represent the mean ± SD (standard deviation).

**Table 3:** Comparison of the amount of individual fatty acids in moringa extract from leaves and seeds with other results [27] (leaves, %).

Compound	Leaves (%)	Seeds (%)	[27] Leaves
myristic (C14:0)	0.11 ± 0.01	0.12 ± 0.01	3.66
palmitic (C16:0)	24.84 ± 0.10	5.98 ± 0.06	11.79
palmitoleic (C16:1)	2.04 ± 0.06	1.37 ± 0.05	0.17
heptadecanoic (C17:0)	0.57 ± 0.04	0.23 ± 0.02	3.19
heptadecenoic (C17:1)	0.03 ± 0.01	0.01 ± 0.01	
stearic (C18:0)	11.38 ± 0.12	4.39 ± 0.05	2.13
oleic (C18:1)	25.01 ± 0.13	76.78 ± 0.20	3.96
linoleic (C18:2)	8.80 ± 0.12	0.62 ± 0.04	7.44
linolenic (C18:3)	24.71 ± 0.21	0.13 ± 0.01	44.57
arachidic (C20:0)	0.94 ± 0.03	2.73 ± 0.04	1.16
eicosenoic (C21:0)	0.44 ± 0.04	2.09 ± 0.04	
behenic (C22:0)	0.72 ± 0.04	5.45 ± 0.06	1.27
lignoceric (C24:0)	0.41 ± 0.02	0.10 ± 0.01	2.91
**saturated fats**	**38.97** ± 0.40	**19.00** ± 0.29	**43.91**
**monounsaturated fats**	**27.52** ± 0.20	**80.25** ± 0.26	**4.48**
**polyunsaturated fats**	**33.51** ± 0.33	**0.75** ± 0.05	**52.21**

Data represent the mean ± SD (standard deviation).

**Table 4:** Dry matter, total phenolics content, and antioxidant activity of plant extracts against hydroxyl and superoxide radicals.

Parameter	Seed-Powder Extract (a)	Leaf-Powder Extract (b)	Crushed-Leaf Extract (c)	*p*
DM (g/L)	20.7 ± 0.14	26.8 ± 0.39	28.0 ± 0.43	*p* < 0.001
Phenols (mg GAE/L)	229.5 ± 34.57	635.6 ± 36.53	437.8 ± 31.9	*p* < 0.001
Phenols (mg GAE/g DM)	11.1 ± 1.67	23.7 ± 1.36	15.6 ± 1.14	*p* < 0.001
POI - Superoxide (%)	−68.84 ± 6.50	92.4 ± 15.62	83.6 ± 8.24	*p* < 0.001
POI - Superoxide (%/g DM)	−3.3 ± 0.31	3.4 ± 0.58	3.0 ± 0.29	*p* < 0.001
POI - Hydroxyl (%)	55.0 ± 3.90	73.1 ± 6.91	60.7 ± 3.82	*p* < 0.001
POI - Hydroxyl (%/g DM)	2.7 ± 0.19	2.7 ± 0.26	2.2 ± 0.14	*p* = 0.023

Data represent the mean ± SD (standard deviation); statistics according ANOVA; GA = gallic acid equivalent.

**Table 5:** Content of analyzed minerals in dried material of *Moringa oleifera* from the Caribbean.

Minerals	Mg	Cu	P	Zn	Fe	Ca
mg/kg	1618.7	5.9	2067.0	30.6	57.7	12,567.0

**Table 6:** Content of analyzed vitamins in dried material of *Moringa oleifera* from Caribbean.

Vitamins	C	B2	A	PP
other names	ascorbic acid	riboflavin	retinol acetate	nicotinamide
mg/kg	3210.0	4.0	10.2	34.0

**Table 7:** Content of analyzed amino acids in dried material of *Moringa oleifera* from the Caribbean.

**Amino acids**	***aspartic acid***	***threonine***	***Serine***
g/kg	23.2	10	6.9
**Amino acids**	***glutamic acid***	***proline***	***Glycine***
g/kg	33.8	11.9	14.0
**Amino acids**	***alanine***	***valine***	***Isoleucine***
g/kg	16.1	15.4	12.5
**Amino acids**	***leucine***	***lysine***	***Arginine***
g/kg	22.9	15.8	21.4
**Amino acids**	***tyrosine***	***phenylalanine***	***Histidine***
g/kg	7.8	14.4	7.1
**Amino acids**	***cystine***	***methionine***	***Tryptophan***
g/kg	3.5	4.3	5.4

**Table 8:** Reaction mixture composition (µL) in the superoxide anion radical.

Test Tube	PB	HX	XO	NBT	Sample, SA
“o”	2550	200	-	50	-
“Xo”	2350	200	200	50	-
“o-SA”	2500	200	-	50	50
“SA”	2300	200	200	50	50
“o-Sx”	2500	200	-	50	50
“Sx”	2300	200	200	50	50

PB = phosphate buffer, HX = hypoxanthine, XO = xanthine oxidase, NBT = nitro blue tetrazolium chloride, SA = sodium salicylate, Sx = sample of extract, x: 1 = powdered seeds, 2 = powdered leaves, 3 = crushed leaves.

**Table 9:** Reaction mixture composition (µL) in the hydroxyl radical scavenging activity determination.

Test Tube	PBS	Fe(II)	H2O2	Sample, GA
“100”	980	10	10	-
“o-GA”	990	10	-	10
“:GA”	970	10	10	10
“o-Sx”	980	10	-	10
“Sx”	970	10	10	10

PBS = phosphate buffer saline, GA = gallic acid, Sx = sample of extract, x: 1 = powdered seeds, 2 = powdered leaves, 3 = crushed leaves.

**Table 10:** Reaction mixture composition (µL) in the total phenolics determination.

Test Tube	Working Solution	DDW	GA (500 mg/L)	Sample
“Blank”	2000	150	-	-
“Standard-GA”	2000	-	150	-
“Sample S1”	2000	-	-	150
“Sample S2”	2000	-	-	150
“Sample S3”	2000	-	-	150

DDW = double distilled water, GA = gallic acid, S = sample of extract: S1 = powdered seeds, S2 = powdered leaves, S3 = crushed leaves.
